# Comparing efficacy of intraarticular single crosslinked Hyaluronan (HYAJOINT Plus) and platelet-rich plasma (PRP) versus PRP alone for treating knee osteoarthritis

**DOI:** 10.1038/s41598-020-80333-x

**Published:** 2021-01-08

**Authors:** Shu-Fen Sun, Guan-Chyun Lin, Chien-Wei Hsu, Huey-Shyan Lin, I.-H.siu Liou, Shin-Yi Wu

**Affiliations:** 1 Department of Physical Medicine and Rehabilitation, Kaohsiung Veterans General Hospital, No 386, Ta-Chung 1st Road, Kaohsiung, 813 Taiwan; 2 National Yang-Ming University School of Medicine, Taipei City, Taiwan; 3 School of Nursing, Fooyin University, Kaohsiung, Taiwan; 4 Department of Internal Medicine, Kaohsiung Veterans General Hospital, Kaohsiung, Taiwan

**Keywords:** Outcomes research, Rheumatology

## Abstract

Intraarticular hyaluronan or platelet-rich plasma (PRP) is widely used in the treatment of knee osteoarthritis (OA). The efficacy of combined hyaluronan with PRP remained inconclusive. This study aimed to investigate the efficacy of combined a single crosslinked hyaluronan (HYAJOINT Plus) and a single PRP versus a single PRP in patients with knee OA. In a prospective randomized-controlled trial, 85 patients with knee OA (Kellgren-Lawrence 2) were randomized to receive a single intraarticular injection of HYAJOINT Plus (3 ml, 20 mg/ml) followed by 3 ml PRP (the combined-injection group, N = 43) or a single injection of 3 ml PRP (the one-injection group, N = 42). The primary outcome was the change from baseline in the visual analog scale (VAS) pain (0–00 mm) at 6 months. Secondary outcomes included The Western Ontario and McMaster Universities Osteoarthritis Index (WOMAC, Likert Scale), Lequesne index, single leg stance test (SLS), use of rescue analgesics and patient satisfaction at 1, 3 and 6 months. Seventy-eight patients were available for the intention-to-treat analysis at 6 months. Both groups improved significantly in VAS pain, WOMAC, Lequesne index and SLS at each follow-up visit (p < 0.001). Patients receiving a single PRP experienced significantly greater improvements in VAS pain than patients receiving combined injections at 1-month follow-up (adjusted mean difference: − 5.6; p = 0.017). There were no significant between-group differences in several of the second outcomes at each follow-up visit, except the WOMAC-pain and WOMAC-stiffness scores favoring the one-injection group at 1 month (p = 0.025 and p = 0.011). However, at 6-month follow-up, the combined-injection group achieved significantly better VAS pain reduction (p = 0.020). No serious adverse events occurred following injections. In conclusion, either combined injections of HYAJOINT Plus and PRP or a single PRP alone was safe and effective for 6 months in patients with Kellgren-Lawrence 2 knee OA. Combined injections of HYAJOINT Plus and PRP achieved better VAS pain reduction than a single PRP at 6 months. The results indicating a long term benefit effect of a combination of HYAJOINT Plus and PRP in a particular subset of patients with moderate knee OA need to be replicated in larger trials.

ClinicalTrials.gov number NCT04315103.

## Introduction

Viscosupplementation with hyaluronan (HA) is a well-established treatment option in knee Osteoarthritis (OA)^1^. HA may provide biological actions including anti-inflammatory, antinociceptive, anabolic effects, and it has been known to stimulate endogenous HA synthesis^1–3^. Intraarticular HA injections are recommended in the professional guidelines for patients who can not be effectively managed with nonpharmacologic treatment and simple analgesics^4^.

There are numerous HA formulations different in their origin, concentration and dosing regimens^1^. Most initial HA preparations are derived from rooster-comb tissue and require 3 to 5 intraarticular injections. Subsequent newer HA products have been engineered to provide durable activity and require fewer injections. For example, HYAJOINT Plus consist of chemically crosslinked HA, resulting in increased viscoelasticity and require only one injection^5,6^. The single injection regimen may represent an attractive alternative, as it may decrease patient time expenditure and discomfort associated with the injection process, and offer potential safety benefits to patients.

Platelet-rich plasma (PRP) is an autologous blood product that mainly contains concentrated platelets and growth factors. The growth factors serve to promote local angiogenesis, modulate inflammation, inhibit catabolic enzymes and cytokines, recruit local stem cells and fibroblasts to sites of damage, and induce healthy nearby cells to manufacture greater numbers of growth factors^7^. PRP has also been shown to increase endogenous HA synthesis^8^. Current evidence indicates that PRP may be able to repair cartilage defects, alleviate symptoms of OA, and increase joint function with acceptable safety and positive outcomes^9,10^. Despite the favorable outcomes reported with PRP injection, there is no clarity in reference to the number and frequency of its use in knee OA. A range of single to multiple injections with weekly to every 3- or 4-week schedule had been reported^11,12^. Several recent researches had shown that one injection of PRP is safe and effective for knee OA^12–14^. The single-injection regimen may be more convenient and has less adverse events than multiple injections. This remains an area important for further investigation.

Intraarticular HA or PRP has each been widely used for the treatment of knee OA for years. Combined HA and PRP seems very promising for knee OA. However, the clinical evidence remains unclear. Based on in previous vitro and animal studies, combining HA and PRP may benefit from their dissimilar biological mechanisms for tissue repair and have potentials to synergistically promote cartilage regeneration, inhibit OA inflammation, and modulate the disease process in OA^15–18^. The purpose of this study was to investigate the efficacy and safety of combined a single crosslinked HA with a single PRP versus a single PRP alone in patients with knee OA.

## Materials and methods

### Study design and participants

This was a prospective, randomized-controlled, observer blinded study with 6-month follow-up done between August 2018 and July 2019. Subjects were recruited through advertisements placed in a rehabilitation department of a university-affiliated tertiary care medical center. The inclusion and exclusion criteria are shown in Table 1. All subjects gave written informed consent before participating in the study. The study was approved by the institutional review board for human investigation of Kaohsiung Veterans General Hospital and followed the Declaration of Helsinki, 1996. The study was registered at ClinicalTrials.gov (NCT04315103) (date of registration 19/3/2020).

**Table 1:** Inclusion and exclusion criteria.

**Inclusion criteria**
Aged 20–75 years
Symptomatic knee OA ≥ 6 months despite conservative treatment such as oral analgesics, NSAIDs and/or physical therapy
Kellgren-Lawrence grades 2 knee OA on radiographs taken within the previous 6 months
Average pain at walking ≥ 30 mm on a 100-mm visual analog scale (VAS)
Radiological evidence of bilateral knee OA was accepted if global pain VAS in the contralateral knee < 30 mm
**Exclusion criteria**
Previous orthopedic surgery on the spine or lower limbs
Disabling OA of either hip or foot
Knee instability, apparent joint effusion or marked valgus/varus deformity
Known allergy to hyaluronan products
Women ascertained or suspected pregnancy or lactating
Intraarticular injections into the knee in the past 6 months
Any specific medical conditions (rheumatoid arthritis, active infections, severe cardiovascular diseases, autoimmune diseases, hemiparesis, neoplasm, etc.) that would interfere with the assessments

*OA* osteoarthritis, *NSAID* nonsteroidal anti-inflammatory drugs, *VAS* visual analog scale.

The study consisted of a screen visit, a baseline visit-during which intraarticular injection was done- and follow-up visits at 1, 3 and 6 months postinjection. At 1 week postinjection, we only phoned participants for safety records. Potential study participants returned for a baseline visit after a 1-week washout period for nonsteroidal anti-inflammatory drugs (NSAIDs) and analgesics. Before randomization, demographic data and baseline assessments were collected.

### Randomization procedures

Enrolled patients were randomized (1:1) to 2 groups. Opaque randomization envelopes with sequentially numbered allocation were generated by a person who was not clinically involved in the study. When a patient consented to the trial, the patient selected one of the envelopes and then was given the allocated injection.

### Intervention

The patients in the one-injection group received a single intraarticular injection of PRP (3 ml). The combined-injection group received one injection of HYAJOINT Plus (3 ml) followed consecutively by a single intraarticular PRP (3 ml). All the injections were done by the same experienced physician using aseptic procedures.

For PRP preparation, specialized platelet concentrate separator containing acid citrate dextrose as anticoagulant and a specific separator gel that harvest platelets and plasma, preventing contamination of red blood cells and leukocytes were used. Approximately 7-mL of venous blood was drawn from each patient and was collected into a PLTenus PLUS Platelet Concentrate Separator (TCM Biotech International Corp., Taiwan). The collected blood was centrifuged at a speed of 500 ~ 1200 rpm for 8 min, then 3 mL of leukocyte-poor PRP was harvested. The platelets count was performed, which is 463.83 ± 75.39 × 10^3^/ul in average. The platelet concentration obtained is approximately 2.4 times greater than the baseline platelet concentration, which is considered to be moderately elevated. The moderately elevated platelet concentration seems to induce optimal biologic benefit, with lower platelet concentrations leading to suboptimal effects and higher concentrations to inhibitory effects on osteoblast activity^19^.

The study device HYAJOINT Plus is produced by microbial fermentation. HYAJOINT Plus has been synthesized by a novel crosslinking process by 1, 4-butanediol diglycidyl ether (BDDE) to create an anti-degraded feature (Appendix A). The carefully controlled crosslinking creates a viscous gel with increased density of HA (2% of HA, 20 mg/ml).

Study blinding was accomplished by having an investigator (blinded to the randomization and treatment) perform all the assessments.

No regular analgesics, glucosamine or chondroitin, or physical therapy for knee were permitted during the study. Acetaminophen (500 mg; maximum daily dose, 4 g) was the only rescue medication allowed for knee pain^5^. Acetaminophen was not permitted during the 24-h period prior to each study visit. Use of rescue medication during the study period was recorded in a patient diary^5^.

Major protocol violations included surgery, initiation of physical therapy, and use of proscribed medications^5^. Patients were said to be noncompliant when they missed any patient visit.

### Outcome measures

The primary outcome was the change from baseline in the VAS pain score at 6 months. The patient rated the average severity of knee pain on knee movement over the previous week on a 0–100 mm VAS (0 = no pain to 100 = worst possible pain)^20^.

Secondary outcome measures included the Western Ontario and McMaster Universities Osteoarthritis Index (WOMAC, Likert Scale), Lequesne index, Single-leg stance test (SLS), use of rescue analgesics and patient satisfaction (Appendix B)^21–24^^.^

### Safety assessment

The safety assessment was based on adverse events reported by the patients and physical findings by the evaluator at each follow-up. It was left to the judgement of the evaluator to decide whether each adverse event was related to the study or not. A serious adverse event was defined as an event that was fatal, life threatening, permanently disabling, or requiring hospitalization^5^.

### Statistical analysis

Based on the Statistical Software Sample Power 3.0 and the statistical method used for the study purpose, repeated measures, between-group F-test, the required sample size was estimated to be 31 participants per group (power = 0.8; alpha = 0.05; number of groups = 2; number of measurements = 4; since there are no preliminary data available, we used Cohen’s effect size f in medium level 0.25 and Cohen’s effect size r of correlation among repeated measures in medium level 0.3). Assuming a 20% dropout rate, the number of participants was increased to 38 per group.

Outcomes were analyzed by intention-to-treat (ITT). The ITT population comprised all patients who received the injection and had at least one post-baseline assessment. ITT analysis was performed using the last observation carried forward method to account for missing data.

All statistical procedures were conducted with the Statistical Package for the Social Sciences (version 12.0; SPSS Inc, Chicago, Illinois). Baseline characteristics were compared using t-tests, Fisher’s exact tests, and chi-square tests. Independent samples one-way analysis of covariance (ANCOVAs) using baseline data of outcome variables as the covariates or Johnson-Neyman analyses were used to compare differences in primary and secondary outcomes at 1, 3, and 6-month follow-ups between the two groups. Changes of primary and secondary outcome measures among baseline, 1, 3, and 6-month follow-ups were assessed using repeated measure one-way analysis of variance (ANOVAs) and Bonferroni post hoc tests. Additionally, independent t-tests were used to compare differences in satisfaction at 1, 3, and 6-month follow-ups between the two groups. Repeated measures one-way ANOVA was used to examine if there was significant difference in satisfaction among 1, 3, and 6-month follow-ups. *P* values of < 0.05 were considered statistically significant.

### Ethical approval

The study was approved by the institutional review board for human investigation and followed the Declaration of Helsinki, 1996. The study was registered at ClinicalTrials.gov (NCT04315103).

## Results

### Patient characteristics

**Figure 1:**
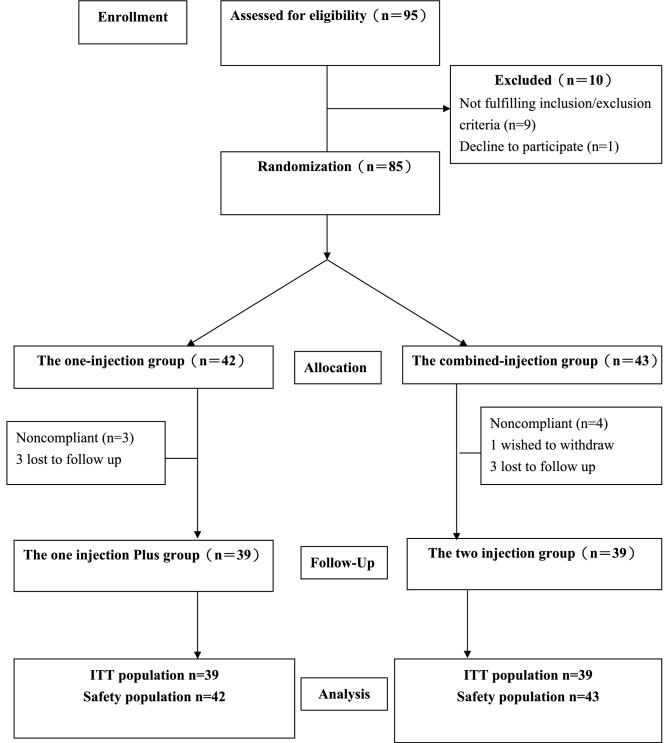
Flow diagram of participants through the trial, *ITT* intention-to-treat.

A total of 95 participants were assessed for eligibility, of whom 85 patients were randomized to either the one-injection group (n = 42) or the combined-injection group (n = 43) (Fig. 1). Seven patients withdrew during the study period, leaving 78 patients available for the ITT analysis at 6-month follow-up. We were able to contact all 7 patients by telephone at the time of the missed follow-up visits, and none of these patients reported an adverse event. There were no significant differences between the 2 groups in demographic and baseline data (p > 0.05) (Table 2).

**Table 2:** Demographic and baseline characteristics of the intention-to-treat population.

Characteristic	One injection (n = 39)	Combined injections (n = 39)	*P* value
Age, years	58.4 ± 8.1	60.6 ± 8.4	0.242^t^
Gender, female (%)	22 (56.4%)	18 (46.2%)	0.365^a^
Weight, kg	65.4 ± 13.3	65.6 ± 14.3	0.932^t^
Height, cm	161.7 ± 6.5	161.6 ± 7.3	0.948^t^
Body mass index, kg/m^2^	24.8 ± 4.1	25.0 ± 4.6	0.873^t^
Employment status (light worker/heavy labor)	37 (94.9%)/2 (5.1%)	35 (89.7%)/4 (10.3%)	0.337^f^
Knee injection side (left/right)	19 (48.7%)/20 (51.3%)	20 (51.3%)/19 (48.7%)	0.821^a^
Disease duration, years	4.4 ± 2.3	5.5 ± 3.4	0.089^t^

The values are given as mean ± the standard deviation or number of patients, with the percentage in parentheses.

^a^χ^2^-test.

^f^Fisher's exact test.

^t^Independent t-test.

*P < 0.05.

### Primary and secondary outcomes

Both groups showed significant improvements in VAS pain, WOMAC (including 3 subscale scores and total scores), Lequesne index scores and SLS among baseline, 1, 3, and 6 months after injections (p < 0.001) (Tables 3 and 4).

**Table 3:** Comparisons of outcomes among groups at different time points and across four waves of times in individual groups.

Outcome	One injection (N = 39)	Combined injections (N = 39)	A.M.D. (95% CI)	*P* value ^a^
**VAS pain baseline**	35.3 ± 16.5	40.0 ± 15.7	− 4.7 (− 12.0,2.5)^d^	0.199^t^
1 month	13.4 ± 13.3	21.9 ± 15.1	− 5.6 (− 10.2,− 1.0)	0.017*
3 month	9.5 ± 13.7	11.9 ± 12.9		Figure 2
6 month	14.9 ± 17.9	8.2 ± 11.4	7.9 (1.3,14.5)	0.020*
*P* Value^b^ (post hoc test)	< 0.001* (B > 1 M = 3 M = 6 M)	< 0.001* (B > 1 M = 3 M = 6 M)		
**WOMAC-pain baseline**	5.6 ± 3.4	6.0 ± 3.2	− 0.4 (− 1.9,1.1)^d^	0.586^t^
1 month	2.7 ± 2.3	3.9 ± 2.9	− 1.1 (− 1.9,− 0.1)	0.025*
3 month	2.2 ± 2.5	3.0 ± 2.3	− 0.7 (− 1.7,0.2)	0.140
6 month	3.1 ± 3.2	3.5 ± 2.7	− 0.2 (− 1.3,1.0)	0.801
*P* Value^b^ (post hoc test)	< 0.001* (B > 1 M = 3 M = 6 M)	< 0.001* (B > 1 M = 3 M = 6 M)		
**WOMAC-stiffness baseline**	2.5 ± 1.3	2.3 ± 1.4	0.2 (− 0.4,0.8)^d^	0.608^t^
1 month	1.1 ± 1.1	1.6 ± 1.1	− 0.6 (− 1.1,− 0.1)	0.011*
3 month	0.9 ± 1.1	1.3 ± 1.1	− 0.4 (− 0.9,0.1)	0.086
6 month	1.3 ± 1.2	1.6 ± 1.1	− 0.4 (− 0.9,0.1)	0.117
*P* Value^b^ (post hoc test)	< 0.001* (B > 1 M = 3 M = 6 M)	< 0.001* (B > 1 M = 3 M = 6 M)		
**WOMAC-function baseline**	19.9 ± 10.5	21.2 ± 12.3	− 1.2 (− 6.4,3.9)^d^	0.635^t^
1 month	11.3 ± 8.5	13.8 ± 9.5	− 1.9 (− 4.8,1.1)	0.209
3 month	8.2 ± 7.9	10.4 ± 9.0	− 1.8 (− 5.2,1.6)	0.285
6 month	12.1 ± 10.4	12.9 ± 11.1	− 0.0 (− 3.5,3.4)	0.998
*P* Value^b^ (post hoc test)	< 0.001* (B > 6 M > 3 M,B > 1 M)	< 0.001* (B > 1 M = 3 M = 6 M)		
**WOMAC-total baseline**	28.0 ± 14.2	29.5 ± 16.2	− 1.5 (− 8.4,5.4)^d^	0.667^t^
1 month	15.0 ± 11.2	19.3 ± 12.8	− 3.5 (− 7.3,0.3)	0.072
3 month	11.2 ± 11.1	14.7 ± 11.6	− 2.9 (− 7.4,1.5)	0.193
6 month	16.5 ± 14.4	18.0 ± 14.1	− 0.6 (− 5.3,4.2)	0.815
*P* Value^b^ (post hoc test)	< 0.001* (B > 6 M > 3 M,B > 1 M)	< 0.001* (B > 1 M = 3 M = 6 M)		

The values are given as mean ± standard deviation.

*VAS* Visual analog scale for pain, *WOMAC* The Western Ontario and McMaster Universities Osteoarthritis Index, *A.M.D.* Adjusted mean difference, *CI* Confidence interval.

^a^Between-group difference determined using independent samples one-way ANCOVA(baseline data as covariate) or Johnson-Neyman technique (Fig. 1).

^b^Within-group difference determined using repeated measures one-way ANOVA.

^t^independent t-test.

*P < 0.05.

**Table 4:** The comparison of outcome measures of Lequesne index and SLS.

	One injection (N = 39)	Combined injections (N = 39)	A.M.D. (95% C.I.)	*p* value^*a*^
**Lequesne index baseline**	8.6 ± 3.5	9.1 ± 4.4	− 0.5 (− 2.3, 1.3)	0.572^t^
1 month	5.6 ± 3.9	6.9 ± 4.6	− 0.9 (− 2.3, 0.5)	0.203
3 month	4.6 ± 3.9	5.6 ± 3.4	− 0.8 (− 2.1, 0.6)	0.254
6 month	5.0 ± 3.9	5.6 ± 3.9	− 0.2 (− 1.6, 1.2)	0.750
*P* Value^b^ (post hoc test)	< 0.001* (B > 1 M > 3 M,B > 6 M)	< 0.001* (B > 1 M = 3 M = 6 M)		
**SLS baseline**	31.9 ± 28.4	27.1 ± 22.8	4.8 (− 6.8, 16.4)	0.413 ^t^
1 month	52.9 ± 46.5	52.3 ± 47.2	− 5.2 (− 21.1, 10.7)	0.518
3 month	54.3 ± 44.0	50.0 ± 37.1	− 0.8 (− 14.6, 13.0)	0.909
6 month	50.4 ± 32.2	46.9 ± 35.1	− 1.0 (− 11.6, 9.5)	0.846
*P* Value^b^ (post hoc test)	< 0.001* (B < 1 M = 3 M = 6 M)	< 0.001* (B < 1 M = 3 M = 6 M)		

The values are given as mean ± standard deviation

*SLS* Single limb stance, *A.M.D.* Adjusted mean difference, *C.I*. confidence interval.

^a^Between-group difference using independent samples one-way ANCOVA (baseline data as covariate).

^b^Within-group difference using repeated measure one-way ANOVA.

^t^Independent t-test.

*p value < 0.05.

**Figure 2:**
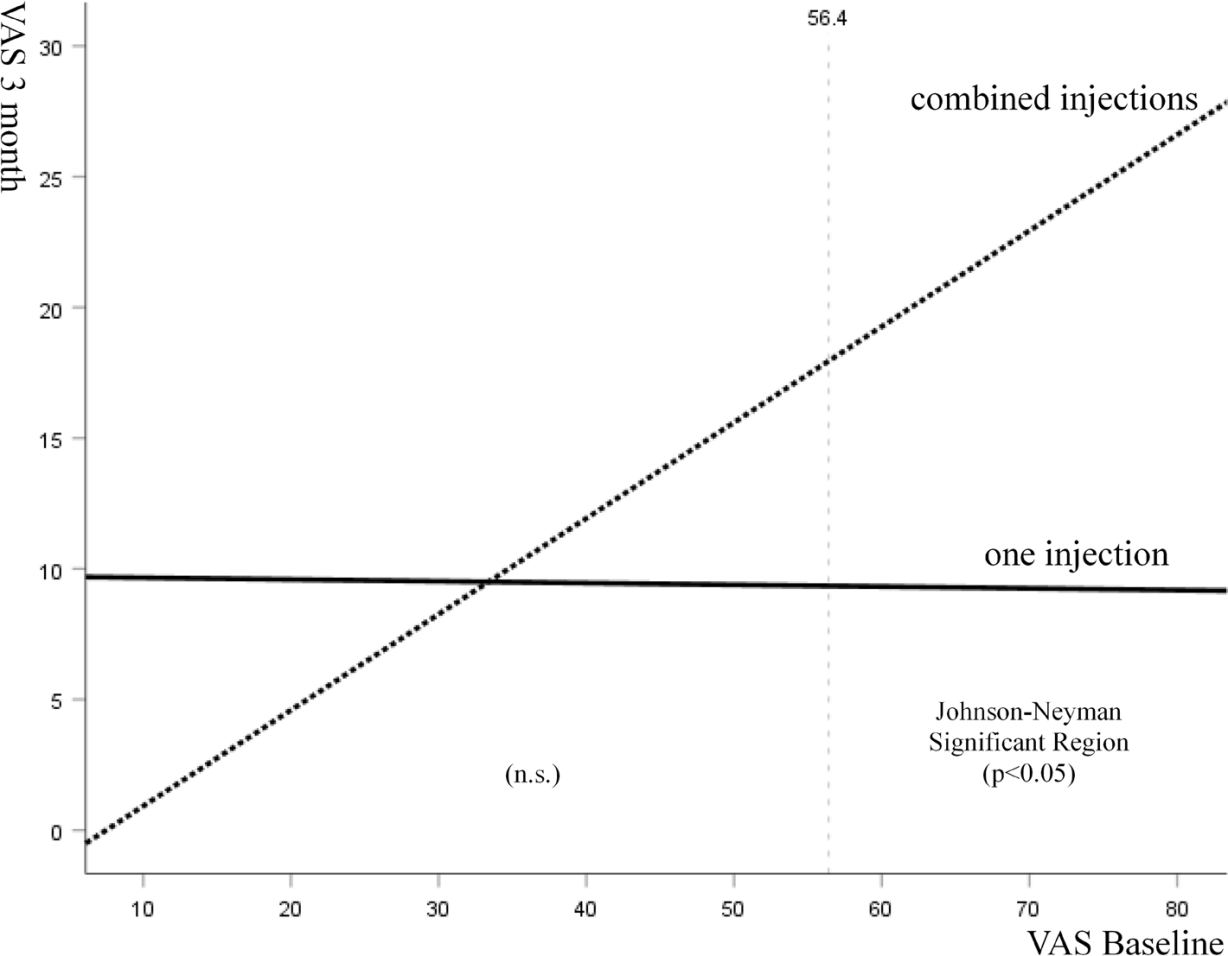
Graph showing that in patients with baseline VAS > 56.4 mm, those treated with one injection experienced a greater reduction in VAS pain than those treated with combined injections at 3-month follow-up.

The mean VAS scores improved by 21.9 mm, 25.8 mm and 20.4 mm from baseline at 1, 3 and 6-month follow-ups in the one-injection group, whereas the VAS scores improved by 18.1 mm, 28.1 mm and 31.8 mm from baseline in the combined-injection group (Table 3). Between-group comparison showed that patients receiving one injection of PRP experienced significantly greater VAS pain reduction than patients receiving combined injections at 1-month follow-up (adjusted mean difference: -5.6; p = 0.017) (Table 3). However, at 6-month follow-up, the combined-injection group achieved significantly better VAS pain reduction than the one-injection group (adjusted mean difference: 7.9; p = 0.020). Additional Johnson-Neyman analyses revealed the region of significance between the two groups at 3 months (Table 3, Fig. 2). In patients with baseline VAS pain > 56.4 mm, those treated with one injection had significantly better VAS pain reduction than combined injections at 3-month follow-up (Fig. 2).

Both groups showed significant improvements from baseline in WOMAC subscale and total scores at 1 month postinjection, and the improvements could maintain at 6 months (Table 3). The between-group differences in WOMAC (including 3 subscale and total scores) were not statistically significant at any follow-up time point, except for significant differences favoring the one-injection group in WOMAC-pain and WOMAC-stiffness subscale scores at 1-month follow-up ((P = 0.025 and P = 0.011, respectively) (Table 3).

The Lequesne index scores improved significantly in both groups (P < 0.001 and P < 0.001, respectively) (Table 4). In the one-injection group, the Lequesne index scores improved significantly from baseline at 1-month postinjection, the maximal improvement was at 3 months, and the effects maintained significant from baseline at 6 months. In the combined-injection group, the Lequesne index scores improved significantly from baseline at 1 month, and the improvements maintained significant at 3 and 6 months. Both groups showed significant improvement in SLS (P < 0.001 and P < 0.001, respectively) at 1-month postinjection and the effects maintained significant from baseline at 3 and 6 months (Table 4). There were no significant between-group differences in Lequesne index scores and SLS at any follow-up time point.

Throughout the study, consumption of analgesics reduced significantly in both groups, with no significant between-group differences (p > 0.05). Patient satisfaction rates were similar, with no significant between-group differences (Table 5). The satisfaction was highest at 3 months postinjection in both groups.

**Table 5:** Patient satisfaction during the study.

Satisfaction	One injection (N = 39)	Combined injections (N = 39)	M.D. (95% C.I.)	*P*^a^
1 month	75.1 ± 22.4	73.0 ± 18.6	2.1 (− 7.2,11.4)	0.657
3 month	78.9 ± 17.6	74.4 ± 14.9	4.5 (− 2.8,11.9)	0.225
6 month	75.5 ± 19.4	72.1 ± 19.6	3.4 (− 5.4,12.2)	0.443
*P* value^b^	0.214	0.541		

The values are given as mean ± the standard deviation

*M.D.* mean difference, *C.I.* confidence interval.

^a^Between-group difference using independent t-test.

^b^Within-group difference using repeated measure one-way ANOVA.

*p value < 0.05.

### Safety outcomes

The safety population comprised all patients who received the injections (N = 85). The frequency and type of adverse events were comparable between the 2 groups. Five patient in the one-injection group (11.9%) and 6 patients in the combined-injection groups (13.9%) developed knee swelling and pain immediately after injection. The knee swelling and pain was mild or moderate, lasting 1 to 3 days, and resolved spontaneously without the need of simple analgesics. No allergic reactions, pseudosepsis or serious adverse events occurred during the study. Adverse events did not lead to study discontinuation in both groups.

## Discussion

This was the first randomized-controlled trial comparing a single intraarticular HA combined with a single PRP versus a single PRP for the treatment of Kellgren-Lawrence 2 knee OA. The study demonstrated that both regimens were safe and effective for 6 months in patients with knee OA. The improvements in VAS pain, WOMAC-pain and WOMAC-stiffness subscale scores were significantly greater in the one-injection group at 1 month. However, the combined-injection group achieved significantly greater VAS pain reduction at 6-month postinjection.

Pain reduction is the primary indication for the use of intraarticular knee injection. In clinical trials of chronic pain treatments, reduction in chronic pain intensity of at least 30% appeared to reflect at least moderate clinically important differences^25^. In this study, the mean VAS pain reduced by 31.8 mm at 6-month follow-up for the combined-injection group (corresponding to a mean reduction of 79.5% from baseline); compared with 20.4 mm (57.8%) reduction from baseline for the one-injection group. It appeared that the magnitude of pain reduction exceeded the clinical meaningful significance. Martini et al. showed a median VAS pain reduction by 21.4 mm (33.3% reduction from baseline) at 6 months after one injection of PRP in patients with Kellgren–Lawrence grade 1 or 2 knee OA^13^. We previously reported a mean VAS pain reduction by 56.2% from baseline at 6 months after a single HYAJOINT Plus injection for patients with Kellgren-Lawrence grade 2 or 3 knee OA^5^. The results of our present study provided evidence that combined HYAJOINT Plus and PRP showed added benefit in pain reduction compared to a single PRP alone. Both HYAJOINT Plus and PRP favor joint repair by different mechanisms, the combination seemed advantageous for the treatment of knee OA.

Based on previous work, the accepted threshold for a minimum clinically important difference (MCID) in WOMAC for OA of the lower limbs is 12% improvement from baseline^26^. In our study, patients in the one-injection group experienced a 41.1% improvement in WOMAC scores from baseline at 6 months, whereas an improvement of 39.0% was noted in the combined-injection group. The improvements in both groups were far greater than the MCID. Previous study by Patel et al. reported a mean WOMAC reduction of 45.5% from baseline at 6 months after a single PRP injection in patients with Ahlback grade 1 or 2 knee OA^12^. Sánchez et al. compared PRP and HA (3 injections on a weekly basis) in patients with knee OA, and they reported that treatment with PRP reduced the WOMAC index by 50%^27^. Direct comparison of the results in these studies was not possible, because of different severity of OA, different PRP preparation and injection regimens.

Lequesne defined effective treatment forms as those leading to a score improvement of 30–40% at the time of follow-up^28^. In our study, the mean Lequesne index scores improved by 3.6 points (41.9% improvement from baseline) in the one-injection group at 6 months, compared with 3.5 points (38.5% improvement from baseline) in the combined-injection group. Both groups reached the criteria used to define treatment effectiveness.

To our knowledge, this was the first study that evaluated SLS after combined HA and PRP injections in patients with knee OA. SLS test is an objective clinical test of standing balance. Balance is a fundamental skill for transfer, walking and many activities of daily living. Pain associated with OA frequently leads to a reduced activity level and weakening of muscles with a secondary increase of instability and joint degeneration. Reduced muscle strength and deficits in lower limb proprioception associated with OA could compromise effective and timely motor responses in maintaining balance^29,30^. The mechanism by which both groups resulted in a clinical benefit on SLS remained unknown, we thought pain reduction was the major reason.

Recent study by Russo et al. showed that PRP addition is not detrimental to the viscosupplementation effect of HA, but HA concentration below 1% is less appropriate for combination with PRP for clinical use in OA^17^. Hence, in our study we chose the novel crosslinked HYAJOINT Plus, with concentration of 2% HA (20 mg/ml; molecular weight > 15 million daltons) to mix with PRP in the combined-injection group.

To date, only limited clinical studies of combined HA and PRP therapy for OA exists^31–35^. Abate et al.treated patients with knee OA using 3 weekly injections of HA combined with PRP, and retrospectively compared results to those treated with PRP alone. They concluded that combined HA and PRP had the same efficacy of PRP alone^31^. Guo et al. compared results of injecting PRP only with HA + PRP (3 weekly injections) in patients with Kellgren-Lawrence grade 1 to 3 knee OA. Similar to the reports by Abate et al., they also concluded that HA + PRP has no differences in outcomes when compared with PRP only^32^. ET Nasser reported that PRP injection appears to improve pain and function in middle-aged women with mild to moderate knee OA, with no added benefit of blending HA with PRP^33^. Dalllari et al. reported in their hip OA study that HA + PRP did not offer any additional benefit over PRP alone at 2, 6 and 12 months^34^. Lana et al. compared HA, PRP and HA + PRP (3 injections, with 2 weeks apart) in patients with Kellgren-Lawrence grade 1 to 3 knee OA, similar to our findings, they reported better results in the combination group^35^. The differences in demographic data, number of injections, different HA/PRP preparation, along with different scales for assessment in various studies might be responsible for the difference in results of HA and PRP combination.

In recent years, an increasing number of studies have focused on the rationality of PRP combined with HA for knee OA. Anitua et al., and Marmotti et al. have recently postulated that PRP in combination with HA may be synergistic, by enhancing the migratory potential of fibroblasts in in vitro studies^18,36^. Studies have shown that the combination of PRP and HA may facilitate the activity of signal molecules such as inflammatory molecules, catabolic enzymes, cytokines and growth factors, thereby playing a positive role in repair the degeneration of cartilage and delay the progression of knee OA^35,37^. This synergistic effect mainly changes the role of inflammatory cytokines in the process of chondrocyte degeneration through specific mediators (CD44, TGF-βRII), thereby promoting cartilage regeneration and inhibiting the inflammatory response^15^. Recent meta-analysis showed that intraarticular injection of PRP combined with HA has a unique advantage in the long-term relief of pain in patients with knee OA, which may suggest that the combination therapy may be a better treatment for patients with long-term knee pain in the future^38^.

The combination of HYAJOINT Plus and PRP injection is an innovation, as no studies using this combination in knee OA exists. In our study, most adverse events were mild and self-limiting, supporting a favorable safety profile of both groups. The results provide evidence that HYAJOINT Plus combined with a single PRP may be a potential novel treatment option for patients with knee OA. However, as we recruited patients with Kellgren-Lawrence grade 2 knee OA only, the results might not be applied to patients with overweight, and with a more painful and advanced stage disease. One interesting finding we identified from this study was that, in patients with baseline VAS pain > 56.4 mm, those treated with a single PRP had significantly better VAS pain reduction than those treated with combined injections at 3-month follow-up. Further studies are needed to identify the characteristics of patients most likely to benefit from combined injection regimens and more predictors of good response have yet to be defined.

Several limitations existed in this study. First, this was a single center study with small sample size, and we recruited patients with Kellgren-Lawrence grade 2 tibiofemoral OA only. The result cannot be generalized to all the OA populations with different radiographic severity. Second, the injector and the patients were not blinded in this study. However, the injector was not involved in outcome assessments and data analyses and the evaluator remained blinded to the study groups and treatment. Third, we did not have a group using a single HA only or a true control group using saline because of budget limits and ethical consideration, thus we could not comment further on synergism between HA and PRP. Forth, the injected volume in the combined-injection group was twofold that of the one-injection group. The higher volume may possibly cause a dilution in the amount of PRP growth factors or by excessive capsular distension that could have implications on the outcomes. Fifth, the combination of HA and PRP in our study was done by sequential injections of HA followed by PRP. Whether PRP should be given first, followed by HA, vice versa or by new technology to mix both as a newer medical device remained unknown. Furthermore, the magnitude of improvement is huge in this trial, suggesting an important placebo effect. We did not investigate the effect of injections on the cartilage and joint structure. Advanced imaging such as MRI may provide more objective data as to the treatment benefit.

In conclusion, this trial showed that either a single intraarticular HYAJOINT Plus combined with a single PRP or a single PRP alone was effective and safe for the treatment of knee OA over 6 months. One injection of PRP was superior to combined injections of HYAJOINT Plus and PRP in VAS pain reduction, WOMAC-pain and WOMAC-stiffness scores improvement at 1 month. Combined injections were superior to one PRP in VAS pain reduction at 6 month. The difference between groups was statistically significant, but limited in absolute value and observed in a subset patients with moderate knee OA (on X rays and on a clinical point of view). Further studies are needed to define the optimal schedule, dosage and ideal concentration of HA to mix with PRP for the treatment of knee OA. The cost-effective values of different combination regimens should be explored also.

## Supplementary Information

Supplementary Information

## References

[1] Colen S, Bekerom MP, Mulier M, Haverkamp D (2012). Hyaluronic acid in the treatment of knee osteoarthritis: A systematic review and meta-analysis with emphasis on the efficacy of different products. BioDrugs..

[2] Moreland LW (2003). Intraarticular hyaluronan (hyaluronic acid) and hylans for the treatment of osteoarthritis: Mechanisms of action. Arthr. Res. Ther..

[3] Lisignoli G (2001). Anti-Fas-induced apoptosis in chondrocytes reduced by hyaluronan: Evidence for CD44 and CD54 (intercellular adhesion molecule 1) involvement. Arthritis. Rheum..

[4] Zhang W (2008). OARSI recommendations for the management of hip and knee osteoarthritis. Part II: OARSI evidence-based, expert consensus guidelines. Osteoarthr. Cartil..

[5] Sun SF (2017). Comparison of single intra-articular injection of novel hyaluronan (HYA-JOINT Plus) with Synvisc-One for knee osteoarthritis. J. Bone Jt. Surg. Am..

[6] Tuan S (2018). Improvement of self-reported functional scores and thickening of quadriceps and femoral intercondylar cartilage under ultrasonography after single intra-articular injection of a novel crosslinked hyaluronic acid in the treatment of the knee osteoarthritis. J. Back Musculoskelet. Rehabil..

[7] Cugat R (2015). Biologic enhancement of cartilage repair: The role of platelet-rich plasma and other commercially available growth factors. Arthroscopy.

[8] Anitua E (2007). Platelet-released growth factors enhance the secretion of hyaluronic acid and induce hepatocyte growth factor production by synovial fibroblasts from arthritic patients. Rheumatology.

[9] Chang KV (2014). Comparative effectiveness of platelet-rich plasma injections for treating knee joint cartilage degenerative pathology: A systematic review and meta-analysis. Arch. Phys. Med. Rehabil..

[10] Zhu Y (2013). Basic science and clinical application of platelet-rich plasma for cartilage defects and osteoarthritis: A review. Osteoarthritis Cartil..

[11] Görmeli G (2017). Multiple PRP injections are more effective than single injections and hyaluronic acid in knees with early osteoarthritis: A randomized, double-blind, placebo-controlled trial. Knee Surg. Sport Traumatol. Arthrosc..

[12] Patel S, Dhillon MS, Aggarwal S, Marwaha N, Jain A (2013). Treatment with platelet-rich plasma is more effective than placebo for knee osteoarthritis: A prospective, double-blind, randomized trial. Am. J. Sports Med..

[13] Martini LI (2017). Single platelet-rich plasma Injection for early stage of osteoarthritis of the knee. Joints..

[14] Buendía-López D, Medina-Quirós M, Fernández-Villacañas Marín MÁ (2018). Clinical and radiographic comparison of a single LP-PRP injection, a single hyaluronic acidinjection and daily NSAID administration with a 52-week follow-up: A randomized controlled trial. J. Orthop. Traumatol..

[15] Andia I, Abate M (2014). Knee osteoarthritis: Hyaluronic acid, platelet-rich plasma or both in association?. Expert. Opin. Biol. Ther..

[16] Chen WH (2014). Synergistic anabolic actions of hyaluronic acid and platelet-rich plasma on cartilage regeneration in osteoarthritis therapy. Biomaterials.

[17] Russo F (2016). Platelet rich plasma and hyaluronic acid blend for the treatment of osteoarthritis: Rheological and biological evaluation. PLoS ONE.

[18] Anitua E, Sanchez M, De la Fuente M, Zalduendo MM, Orive G (2012). Plasma rich in growth factors stimulates tendon and synovial fibroblast migration and biological properties of HA. Knee Surg. Sports Traumatol Arthrosc..

[19] Weibrich G, Hansen T, Kleis W, Buch R, Hitzler WE (2004). Effect of platelet concentration in platelet-rich plasma on peri-implant bone regeneration. Bone.

[20] Huskisson EC (1974). Measurement of pain. Lancet.

[21] McConnell S, Kolopack P, Davis AM (2001). The Western Ontario and McMaster Universities Osteoarthritis Index (WOMAC): A review of its utility and measurement properties. Arthritis. Care Res..

[22] Bellamy N, Buchanan WW, Goldsmith CH, Campbell J, Stitt LW (1988). Validation study of WOMAC: A health status instrument for measuring clinically important relevant patient outcomes to antirheumatic drug therapy in patients with osteoarthritis of the hip or knee. J. Rheumatol..

[23] Lequesne MG, Mery C, Samson M, Gerard P (1987). Indexes of severity for osteoarthritis of the hip and knee: Validation-value in comparison with other assessment tests. Scand. J. Rheumatol. Suppl..

[24] Bohannon RW, Larkin PA, Cook AC, Gear J, Singer J (1984). Decrease in timed balance scores with aging. Phys. Ther..

[25] Dworkin DH (2008). Consensus statement: Interpreting the clinical importance of treatment outcomes in chronic pain clinical trials: IMMPACT recommendations. J. Pain..

[26] Angst F, Aeschlimann A, Stucki G (2001). Smallest detectable and minimal clinically important differences of rehabilitation intervention with their implications for required sample sizes using WOMAC and SF-36 quality of life measurement instruments in patients with osteoarthritis of the lower extremities. Arthritis. Rheum..

[27] Sánchez M (2012). A randomized clinical trial evaluating plasma rich in growth factors (PRGF-endoret) versus hyaluronic acid in the short-term treatment of symptomatic knee osteoarthritis. Arthroscopy.

[28] Lequesne MG (1997). The algofunctional indices for hip and knee osteoarthritis. J. Rheumatol..

[29] Hassan B, Mockett S, Doherty M (2001). Static postural sway, proprioception, and maximal voluntary quadriceps contraction in patients with knee osteoarthritis and normal control subjects. Ann. Rheum. Dis..

[30] Fisher NM, Pendergast DR (1997). Reduced muscle function in patients with osteoarthritis. Scand. J. Rehabil. Med..

[31] Abate A, Verna S, Schiavone C, Di Gregorio P, Salini V (2015). Efficacy and safety profile of a compound composed of platelet-rich plasma and hyaluronic acid in the treatment for knee osteoarthritis (preliminary results). Eur. J. Orthop. Surg. Traumatol..

[32] Guo Y (2016). Treatment of knee osteoarthritis with platelet-richplasma plus hyaluronic acid in comparison with platelet-rich plasma only. Int. J. Clin. Exp. Med..

[33] Nasser ET (2018). Treatment of knee osteoarthritis with platelet-rich plasma in comparison with platelet-rich plasma plus hyaluronic acid: A short-term double-blind randomized clinical study. Egypt Orthop J..

[34] Dallari D (2016). Ultrasound-guided injection of platelet-rich plasma and hyaluronic acid, separately and in combination for hip osteoarthritis: A randomized controlled study. Am. J. Sports Med..

[35] Lana JF (2016). Randomized controlled trial comparing hyaluronic acid, platelet-rich plasma and the combination of both in the treatment of mild and moderate osteoarthritis of the knee. J. Stem Cells Regen. Med..

[36] Marmotti A (2012). One-step osteochondral repair with cartilage fragments in a composite scaffold. Knee Surg. Sports Traumatol. Arthrosc..

[37] Saturveithan C (2016). Intra-articular hyaluronic acid (HA) and platelet rich plasma (PRP) injection versus hyaluronic acid (HA) injection alone in patients with grade III and IV knee osteoarthritis (OA): A retrospective study on functional outcome. Malays. Orthop. J..

[38] Zhao J (2020). Effects and safety of the combination of platelet-rich plasma (PRP) and hyaluronic acid (HA) in the treatment of knee osteoarthritis: A systematic review and meta-analysis. BMC Musculoskelet. Disord..

