# Supplementary material for: Comparing efficacy of intraarticular single crosslinked Hyaluronan (HYAJOINT Plus) and platelet-rich plasma (PRP) versus PRP alone for treating knee osteoarthritis
Source: Sci Rep. 2021 Jan 8;11:140. doi: 10.1038/s41598-020-80333-x (PMC7794411; doi:10.1038/s41598-020-80333-x)
Supplement: Supplementary file 1 — Supplementary Information [file 41598_2020_80333_MOESM1_ESM.doc]

**Comparing efficacy of intraarticular single crosslinked Hyaluronan (HYAJOINT Plus) and platelet-rich plasma (PRP) versus PRP alone for treating knee osteoarthritis**

Shu-Fen Sun,^1, 2^ MD, Guan-Chyun Lin, PhD ^3^, Chien-Wei Hsu,^2, 4^ MD, Huey-Shyan Lin,^3^ PhD, I-Hsiu Liou^1^, Shin-Yi Wu,^1^ MD

Department of Physical Medicine and Rehabilitation, Kaohsiung Veterans General Hospital^1^; National Yang-Ming University School of Medicine, Taiwan^2^; School of Nursing, Fooyin University, Taiwan^3^; Department of Internal Medicine, Kaohsiung Veterans General Hospital^4^

**Running Title: Combined Intraarticular HYAJOINT Plus with a Single PRP in Knee Osteoarthritis**

Corresponding Author: Dr. Shu-Fen Sun

Address: Department of Physical Medicine and Rehabilitation, Kaohsiung Veterans General Hospital, No 386, Ta-Chung 1^st^ Road, Kaohsiung 813, Taiwan

Tel: 886-73422121-4210

Fax: 886-73420243

E-mail: [sfsun.tw@yahoo.com.tw](mailto:sfsun.tw@yahoo.com.tw)

Total number of pages: 39

**Appendix A**

1. HYAJOINT Plus crosslinking

The crosslinker of HYAJOINT Plus is 1, 4-butanediol diglycidyl ether (BDDE). The eposide groups in BDDE link to the primary hydroxyl groups in the hyaluronic acid through an ether bond or to hydrolyze into an alcohol (Figure 1).


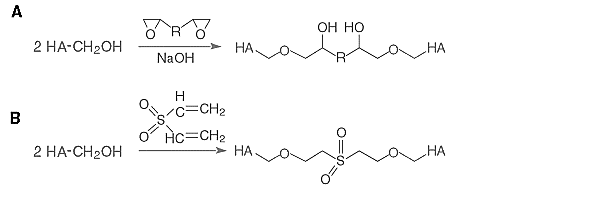


**Appendix B**

Description of the secondary outcome measures:

The Western Ontario and McMaster Universities Osteoarthritis Index (WOMAC, Likert Scale) is a 24-item questionnaire with 3 subscales measuring pain, stiffness, and physical function. Total score is 96 and higher scores indicate worse outcomes.

**Lequesne index** was used to assess severity of knee symptoms during the last week. **It** includes the measurement of pain, walking distance, and activities of daily living. Maximal score is 24 and higher scores represent worse function.

Single-leg stance test (SLS) is done by raising one foot up without touching it to the supported lower extremity with target knee and maintain balance for as long as possible. Each participant performed 3 trials, and the best result of the 3 trials was recorded.

Patients were asked to rate their treatment satisfaction compared to the preinjection condition, using a 100 mm VAS (0= completely dissatisfied, 100=completely satisfied).
